# The Best Dentist’s Chair

**Published:** 1878-08

**Authors:** 


### Editorial.

### THE DENTIST'S OPERATING CHAIR.

Of all the implements and appliances used by the dentist there is none perhaps more important than the operating chair. If good, it ministers to the ease and efficiency of the operator, and much to the comfort and satisfaction of the patient.

Too little attention is given by the majority of operators to the material points of the chair.

Nearly all the chairs as yet furnished to the profession have been more or less defective in many points. Many are unnecessarily bulky and clumsy, and often entirely wanting in some important movement, and even those they have are exceedingly defective. The capacity of the chair should be such that the average patient would fill it. It is unfortunate to have a chair so large that the ordinary patient can slip and slide about in it. The width of the seat between the arms should not be more than eighteen inches, and it would be well if the arms were adjustable, that they might be brought within twelve inches of each other. The depth of seat from front to back should not be more than sixteen inches.

The back should be as narrow as admissible, so that under no circumstances would it be in the least in the way of the operator. The seat of the chair when at its lowest point should be about twenty inches from the floor, and thirty-two at the highest. The body of the chair should rotate, at least to the extent of two-thirds of a circle. There should also be the reclining movement, so that the occupant of the chair may be placed at any angle from the erect position to almost the horizontal. The mechanism should be such that these movements could be easily and conveniently operated; even when a patient is on the chair, and definitely in the control of the operator. The back should have an upward and downward movement of from eight to nine inches, and susceptible of being firmly fixed at any point. It should also be adjustable for adaptation to the patient. The head-rest should have a universal movement. It, with its attachments, should be so constructed that it can be readily fixed in any position desired. It should have a lateral movement of from six to eight inches, a forward and backward movement of from three to four inches, and should be susceptible of a vertical and horizontal position, and of any inclination between these. The foot-board should have an upward and downward movement of at least seven inches, and should be so arranged as to be readily adjusted and firmly fixed for either the tallest or shortest person. A lateral inclining movement of the body of the chair has been regarded as important by some, but it is rarely called for, and the disadvantages that arise from it far more than balance any benefit to be gained by it. The base of the chair should always be of such form and weight as to sit perfectly firm under any weight and movement to which the chair may be subjected. It should never be fastened to the floor nor put upon casters. The above is an outline of the principal features of a good, practicable dentist's chair.

A very large proportion of the chairs offered to the profession are wanting in more or less of these qualities; and many of them in some of the most important ones. Others have their machinery so constructed that it requires about one horse power to work them, and sometimes they break away from the worker. Every chair should be so made that any of its movements may be operated while an occupant is on the seat, and especially is this true of the movements that change the position of the patient. The position of the patient is frequently to be changed, first, to give easy and the most direct access to the point to be operated upon; and in the second place change of position is often necessary for the comfort of both the patient and operator. All other things being equal a better operation will be made upon a good chair than upon a poor one.

As a rule our best operators have the best appliances throughout, and the poorest have the worst. As in respect to almost everything else, the best dental chairs are expensive; at least they seem to be open to that criticism to those of us who are impecunious; and were it practicable it would be much to the interest of the profession and to those who are patients as well, if a chair combining all the good qualities could be furnished for from sixty to eighty dollars. Perhaps when the multitude of patents that now attach to them, become obsolete, something in this direction may be realized, till then let us possess our souls in patience.

